# Home Oral Care of Periodontal Patients Using Antimicrobial Gel with Postbiotics, Lactoferrin, and Aloe Barbadensis Leaf Juice Powder vs. Conventional Chlorhexidine Gel: A Split-Mouth Randomized Clinical Trial

**DOI:** 10.3390/antibiotics11010118

**Published:** 2022-01-17

**Authors:** Andrea Butera, Simone Gallo, Maurizio Pascadopoli, Damiano Taccardi, Andrea Scribante

**Affiliations:** 1 Unit of Dental Hygiene, Section of Dentistry, Department of Clinical, Surgical, Diagnostic and Pediatric Sciences, University of Pavia, 27100 Pavia, Italy; damiano.taccardi01@universitadipavia.it; 2 Unit of Orthodontics and Pediatric Dentistry, Section of Dentistry, Department of Clinical, Surgical, Diagnostic and Pediatric Sciences, University of Pavia, 27100 Pavia, Italy; simone.gallo02@universitadipavia.it (S.G.); andrea.scribante@unipv.it (A.S.)

**Keywords:** dentistry, periodontitis, scaling and root planing, non-surgical periodontal therapy, probiotics, postbiotics, chlorhexidine, periodontology, randomized clinical trial

## Abstract

Periodontitis is a progressive destruction of both soft and hard tooth-supporting tissues. In the last years, probiotics have been proposed as a support to the gold standard treatment scaling and root planing (SRP), but no extensive literature is present as regards the effect of the more recent postbiotics. Thirty patients subjected to SRP were randomly assigned to two domiciliary hygiene treatments based on the following oral gels: the postbiotics-based Biorepair Parodontgel Intensive (Group 1) and the chlorhexidine-based Curasept Periodontal Gel (Group 2). At baseline (T_0_) and after 3 and 6 months (T_1_–T_2_), the following periodontal clinical parameters were recorded: Probing Pocket Depth (PPD), recession, dental mobility, Bleeding on Probing (BoP), and Plaque Control Record (PCR). A significant intragroup reduction was assessed in both groups for PPD, BoP, and PCR; conversely, recession significantly increased in both groups, whereas dental mobility did not vary. As regards intergroup comparisons, no statistically significant differences were assessed. Both gels, respectively, containing antioxidant natural ingredients and chlorhexidine, are effective for the domiciliary treatment of periodontitis. Further studies are required to evaluate the singular chemical compounds of the gels expected to exert the beneficial action assessed in this preliminary study.

## 1. Introduction

Periodontitis is an inflammatory condition that involves both soft and hard tooth-supporting tissues and constitutes the major cause of tooth loss after dental decay. This process is the result of an untreated gingival inflammation linked to bacterial plaque accumulation. The destruction of the tissues is due both to a direct mechanism, exerted by the pathogenetic action of bacteria by means of their virulence factors, as well as to an indirect mechanism deriving from the excessive inflammatory reaction of the host causing the release of chemical mediators, which further promotes tissue damage [1]. From a clinical point of view, this results in a bleeding of the marginal gum, and, subsequently, in an irreversible periodontal attachment loss with the formation of pockets and recessions, bone loss, dental mobility, and exfoliation [2].

The factors mostly related with periodontal disease are smoking [3], the alteration of leukocytes [4], immunosuppression [5], diabetes [5], and genetic polymorphisms of genes related to the production of inflammatory cytokines [6]. However, bacterial plaque accumulation is actually the most important risk factor, since specific bacteria, called periodontopathogens, cause an inflammatory response which might lead itself to tissue destruction factors [7].

Considering the therapeutic approach, it is based on the removal of the bacterial biofilm, avoiding further tissue damage. Scaling and root planing (SRP) is the gold standard non-surgical therapy based on the removal of dental plaque and calculus (scaling), as well as on smoothing the root surfaces (root planing) [8]. In spite of its efficacy, bacterial recolonization following the treatment is the most represented shortcoming of SRP [9]. On the basis of this consideration, further therapeutic approaches have been proposed as adjuncts, like the use of antibiotics [10], the photodynamic treatment [11], ozone application [12], and, in recent years, probiotic therapy [13,14]. As regards these last ones, they have been used more and more in recent years for the treatment of periodontal conditions because of the avoidance of the side effects related to the conventional antibiotic therapy [15]. The Food and Agriculture Organization (FAO) and the World Health Organization (WHO) define probiotics as “live microorganisms which when administered in adequate amounts confer a health benefit on the host” [16]. Probiotics are supposed to play a beneficial role thanks to the competition with pathogens for food and adhesion sites, the production of antimicrobial substances, immunomodulation, and enhancement of the mucosal barrier function [17].

In the scenario of “biotic” agents, a subsequent formulation which has been proposed is represented by postbiotics. These latter include any microbial fermentation product released by, or produced through, the metabolic activity of the microorganism, able to exert a direct or indirect beneficial effect on the host, specifically an antioxidant action [18].

The aim of this randomized clinical trial is to compare the efficacy of two different domiciliary oral gels (respectively, the former with a postbiotic content and the latter a chlorhexidine-based one), in addition to SRP, in improving periodontal clinical indexes. The statistical null hypothesis of the study is that there are no significant intra- and intergroup differences in clinical indexes considering the different timepoints. 

## 2. Results

### 2.1. Participant Flow and Baseline Data

**Figure 1:**
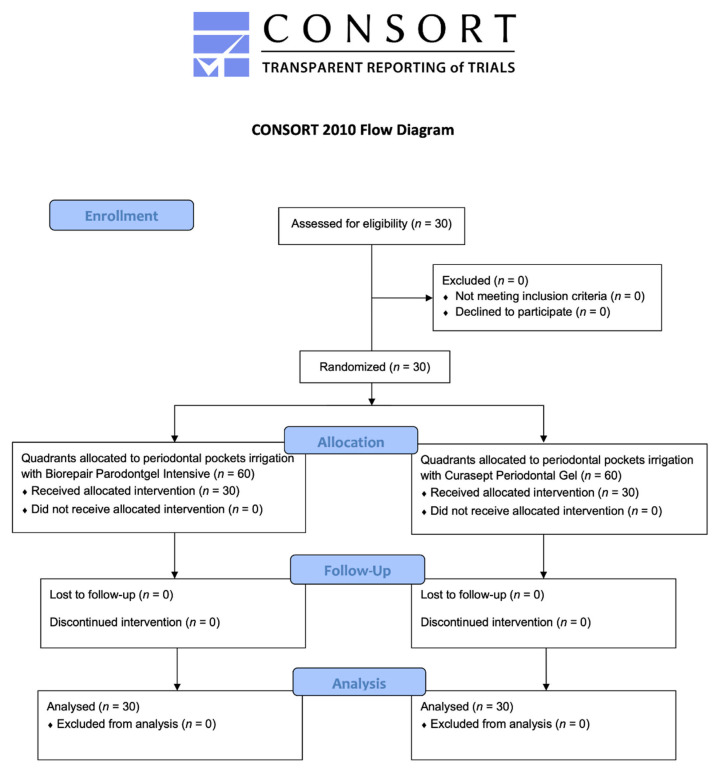
Flow chart of the study.

Thirty patients responding to the inclusion criteria were asked to participate to the study. They all agreed to participate and received the allocated interventions. No patient was excluded from the analysis. The flow chart of the study is shown in Figure 1. At the baseline, the sample showed a mean age of 51.6 ± 12.6 years (11 females, mean age 54.2 ± 11.3, 19 males, mean age 49.9 ± 13.4). 

The mean values obtained for each variable are shown in the following sections. Intergroup and intragroup comparisons are presented using letter-based comparisons, which provide the assignment of the same letter/letters to groups with not significantly different means. Accordingly, for pairwise comparisons, the presence of the same letter/letters for the means compared shows that no significant differences are present between them [19]. 

### 2.2. Probing Pocket Depth (PPD) 

The PPD values significantly decreased at the end of the study for both the gels (*p* < 0.05). As regards intragroup differences, a significant reduction can be noticed in the Biorepair group at T2, while, for the Curasept group, it was assessed at T3. As regards intergroup differences, no statistically significant difference was found between the two treatments administered (*p* > 0.05) (Table 1).

### 2.3. Recession (R)

The recession values significantly increased in both the groups from T0 to T3 (*p* < 0.05). In the test group, a significant increase was already found at T2 (*p* < 0.05). As regards intergroup differences, no statistically significant difference was found between the two treatments administered (*p* > 0.05) (Table 2).

### 2.4. Dental Mobility

As regards dental mobility, no statistically significant reduction was detected concerning intra- and intergroup differences (*p* > 0.05) (Table 3).

### 2.5. Bleeding on Probing (BoP%)

Concerning BoP, significantly lower values were found at T3 if compared to T0 (*p* < 0.05). This result is more evident in the test group, as a significant intragroup reduction appears from T0 to T1, while, in the control group, it appears at T2. No significant intergroup differences were assessed at any time frame (*p* > 0.05) (Table 4). 

### 2.6. Plaque Control Record (PCR%)

The PCR values significantly decreased from T0 to T3 in both groups (*p* < 0.05). Significant differences can be found between T0 and T1 in the test group, while, for the control group, between T0 and T2 (*p* < 0.05). No significant intergroup differences were assessed at any time frame (*p* > 0.05) (Table 5).

## 3. Discussion

Oral infections represent a frequent event in dentistry [20,21]. Besides dental decay, even tooth-supporting can be affected by pathogenic microorganisms that cause dysbiosis, finally leading to periodontitis [22,23,24]. Although SRP is the most important treatment for periodontitis, many shortcomings, like bacterial recolonization, are linked with this therapeutic action; accordingly, adjunctive therapies are strongly recommended. Among the ones proposed until now, probiotics are the most recent technology and consist of specific bacteria with positive effect on the health; the most representative is the avoidance of antibiotics’ side effects [25]. Symbiotic bacteria are able to induce a modulation of the local environment in order to allow SRP to be efficient [26].

Until now, few studies have tested the action of probiotics on periodontal health. In particular, the most evaluated strain is *Lactobacillus*. For example, *Lactobacillus reuteri (L. reuteri)*, administered in the form of a powder, was shown to improve all clinical periodontal parameters with no differences with respect to amoxicillin [26]. Further studies have confirmed the benefits of *L. reuteri* compared to SRP alone; this is linked to a decrease of proinflammatory cytokines and of the amount of periodontopathogens [26,27,28]. 

In the scenario of “biotics”, a subsequent formulation of the abovementioned probiotics is represented by postbiotics. These latter substances include any microbial fermentation product released by or produced through the metabolic activity of the microorganism, able to exert a direct or indirect beneficial effect on the host [18]. Due to the lack of studies evaluating postbiotics in periodontology, the goal of this randomized clinical trial is to analyze the adjuvant efficacy of a new antioxidant postbiotics-based gel in addition to SRP in improving periodontal clinical indexes. 

The statistical null hypothesis of the study was partially accepted. As regards intragroup differences, a progressive statistically significant decrease was assessed, considering both the products, for the probing pocket depth (PPD), bleeding on probing (BoP%), and plaque control record (PCR%). Conversely, as expected, both the products significantly increased recession (R), which is the common clinical outcome following non-surgical periodontal therapy. As regards dental mobility, no significant intergroup differences were assessed for either of the products, which could be justified by the relatively short follow-up of the study.

On the basis of these results, both the antioxidant postbiotic-based gel and the chlorhexidine-based one appear as a valuable tool for the management of periodontal disease. However, whereas the efficacy of chlorhexidine has been extensively investigated and is widely recognized, the one of postbiotics in dentistry has been poorly addressed to date.

Chlorhexidine (CHX) is one of the most common antiseptic agents in dentistry. It has a broad-spectrum antibacterial activity and it able to reduce plaque, gingival inflammation, and bleeding; it is considered as an adjuvant tool to mechanical oral hygiene procedures and is available in different forms, including mouthwash, gel, aerosol, spray, and disks [29]. Despite its efficacy, several shortcomings are linked to the use of chlorhexidine, including teeth discoloration and dysgeusia. Additionally, the risk of bacterial recolonization is a frequent event. Accordingly, the introduction of new bioactive biomolecules, like “biotics”, is compulsory. The results of the present study show that postbiotics, thanks to their antioxidant effect, are at least as effective as conventional chlorhexidine. Due to their recent introduction, no extensive literature is present. A recent in vitro study was one by Ishikawa et al. [30], who tested cell-free pH-neutralized supernatants (CFS) of *Lactobacillus rhamnosus* Lr32, *L. rhamnosus* HN001, *Lactobacillus acidophilus* LA5, and *L. acidophilus* NCFM against a fimbriated clinical isolate of the Aa JP2 genotype on biofilm formation for 24 h and early and mature preformed biofilms (2 and 24 h). According to the authors, postbiotics deriving from lactobacilli, especially LA5, were able to reduce the colonization levels of Aa and to modulate the expression of virulence factors implicated in the evasion of host defenses. 

To the best of our knowledge, no previous study has been conducted in vivo. Only a recent study was conducted on rats by Park et al. [31]. The authors reported the development of postbiotics with whey bioconversion product produced by *Enterococcus faecalis* M157 KACC 81148BP and mixed whey bioconversion products produced by *E. faecalis* M157 KACC 81148BP and *Lactococcus lactis ssp. lactis* CAU2013 KACC 81152BP. The aim of their report was to test the effect of these biotics for the treatment of periodontal disease and the improvement of gut health. The products were administered orally to PD-induced rats for 8 weeks. According to the results of the study, periodontal lesions were reduced in case of previous treatment with postbiotics, with respect to the control treatment. The bone loss volumes and cytokine levels were lower as well. The authors concluded that the whey bioconversion product produced by *E. faecalis* M157 KACC 81148BP and mixed whey bioconversion products produced by *E. faecalis* M157 KACC 81148BP and *L. lactis* CAU2013 KACC 81152BP were effective on relieving periodontitis and improving the gut health.

Due to the absence of analog studies, no direct comparisons can be done for the results obtained in the current study, aimed at evaluating the efficacy of antioxidant postbiotics contained in a gel formulation. Anyway, the efficacy of both chlorhexidine and postbiotics can be justified by the fact that the former is an antiseptic agent reducing the bacterial amount, whereas postbiotics can act as antiseptics as well but also as antioxidant and immunomodulant-promoting, even an anti-inflammatory effect on the periodontal tissues. Therefore, it might be assumed that the use of Biorepair Parodontgel Intensive is able to lead to a prolonged effect, with respect to the conventional chlorhexidine-based gel considered as the control. It is also important to notice that chlorhexidine should be used for a maximum of 15 days (with a worsening of periodontal indexes at the end of the period), differently from postbiotics. In addition to the effect on soft tissues, it is also remarkable that Biorepair Parodontgel Intensive contains biomimetic Zn-Carbonate Hydroxyapatite, which is recognized for its significant effect on tooth remineralization [14]. Therefore, while chlorhexidine has acted only as an antimicrobial agent, the experimental product here considered might have caused multiple effects on both soft and hard tissues. In fact, the presence of biomimetic Zn-Carbonate Hydroxyapatite might have induced a remineralization of the radicular surface of the teeth; in addition to that, antioxidant postbiotics are likely to have induced an anti-inflammatory and regenerative effect on periodontal tissues, with a repositioning of the junctional epithelium on a re-mineralized radicular surface.

Previously studies have assessed the antioxidant effect of postbiotics with the reduction of oxygen reactive species, like superoxide [18,32,33]. Future research should focus on the evaluation of biochemical parameters in order to confirm with laboratory studies the antioxidant power of the postbiotic gel tested. Moreover, in future studies, it would be interesting even to consider a control group not exposed to the gel treatment. Another concern of the study might be represented by the fact that an only instructed operator assessed the clinical outcomes only once; despite this procedure being chosen in order not to submit the patients to the same procedure more than once, future studies should be designed considering the intra/inter-examiner error.

As to the limitations of the study, we must consider the fact that split-mouth designs could not be the best protocols to evaluate the efficacy of different types of oral gel administered contemporary. In particular, the substantivity of chlorhexidine could cause the contamination of other sites, with the possible combination with the experimental one, thus justifying the absence of intergroup differences assessed in this study. As well, these latter could also be due to possible mistakes of the patients during the domiciliary applications. Moreover, the experimental product contains *Lactobacillus ferment*, which is only one out of 28 ingredients in total. Moreover, its concentration is patent pending, and it has not been released by the manufacturer; accordingly, it could be even very low, as far as we know. It should also be considered that positive or negative interactions might occur between the several ingredients contained in the experimental gel. In particular, there are many additional ingredients that might also be involved in the activity measured, such as lactoferrin (an established anti-inflammatory and antimicrobial peptide but not of postbiotic origin), Aloe Barbadensis Leaf Juice Powder (a protective and anti-inflammatory substance), or Sodium Myristoyl Sarcosinate (a tenside). As regards lactoferrin, its anti-inflammatory action has been studied by means of in vitro periodontitis models, as well as in clinical studies: lactoferrin shows strong in vitro anti-inflammatory properties against gingival fibroblasts infected with *Prevotella intermedia*, whereas the topical administration to patients suffering from periodontitis is able to decrease both the levels of cytokines in crevicular fluid (e.g., IL-6), as well as edema, bleeding, pocket depth, and gingival and plaque index, with a positive variation of clinical attachment levels [34,35,36]. Focusing on Aloe Barbadensis Leaf Juice Powder, several clinical studies have demonstrated that *Aloe* mouthwash and gel are effective in the prevention and treatment of gingivitis and periodontitis by reducing gingival index, plaque index, and probing depth and by increasing bone fill and regeneration [37,38,39,40]. Additionally, *Aloe vera* has proven to be as effective as the other usual treatments, such as chlorhexidine, alendronate, and chlorine dioxide [41].

On the basis of these considerations, and due to the absence of similar studies and to the limited follow-up of the present one, no final conclusions can be done for the use of postbiotics in periodontology. Further studies are required, and, in particular, the effect of postbiotics and chlorhexidine should be assessed not only on the marketed product (because of the content of other ingredients that could bias the results) but, particularly, on the specific singular chemical compounds in order to allow a direct comparison with no confounding factors. 

## 4. Materials and Methods

### 4.1. Trial Design

This is a single-center, split-mouth randomized controlled trial with a 1:1 allocation ratio, approved by the Unit Internal Review Board (registration number: 2021-0210), and registered on Clinicaltrials.gov (NCT number: NCT04781478) at the following link: https://clinicaltrials.gov/ct2/show/NCT04781478?term=NCT04781478&draw=2&rank=1 (accessed on 16 January 2022).

### 4.2. Participants

Patients referring to regular dental hygiene appointments at the Unit of Dental Hygiene, Section of Dentistry, Department of Clinical, Surgical, Diagnostic and Paediatric Sciences of the University of Pavia (Pavia, Italy) were recruited in March 2021 after signing the informed consent. The study lasted until October 2021. Both the interventions and outcomes assessment were conducted at the same Unit. 

The inclusion and exclusion criteria are shown in Table 6.

### 4.3. Interventions and Outcomes

At the baseline (T0), patients were asked to sign the informed consent to participate to the study. After that, the following periodontal indices were collected by an instructed operator using a probe (UNC probe 15; Hu-Friedy, Chicago, IL, USA): BoP (%) (percentages of sites bleeding at probing with respect of total dental sites) [43], PPD (distance between soft margin of the gum and the base of the pocket), gingival recession (distance between enamel-cement junction and soft margin of the gum), PCR (%) (percentage of sites with plaque with respect of total dental sites) [44], and dental mobility [45]. Then, a professional supragingival and subgingival oral hygiene was conducted using a piezoelectric instrument (Multipiezo, Mectron S.p.a, Carasco, Italy) and Gracey curettes (Hu-Friedy, Chicago, IL, USA), followed by supragingival and subgingival application of a decontaminating glycine powder (Glycine Powder, Mectron S.p.a., Carasco, Italy). 

Using a split-mouth design, patients from Group 1 received the application of Biorepair Parodontgel Intensive gel in periodontal pockets of quadrants Q1 and Q3, while, for quadrants Q2 and Q4, Curasept Periodontal Gel was used. For Group 2, the quadrants were inverted. The sides were allocated using random number tables. In Table 7 are shown the two gels used for the study. 

After performing SRP, each site was rinsed, air-dried, and isolated with cotton rolls before applying the gel assigned. After two minutes from their application, each patient was released. 

Patients were visited after 1 (T1), 3 (T2), and 6 (T2) months, and the same procedures were repeated. Together with the in-office part, a domiciliary protocol was conducted by the patients, as, after each visit, they used the two gels in the same quadrants for the following 14 days twice a day (according to the protocol recommended for chlorhexidine). In particular, for the domiciliary self-applications, two different syringes with a plastic blunted needle of 5 to 6 mm in diameter were given to the patients. 

The protocol of the study is shown in Table 8.

A single instructed operator performed the clinical outcomes, and considering the standardization of the clinical procedure, the inter-examiner error was not measured. Due to the uneticity of not treating periodontal patients, it was not technically feasible to consider a negative control group in the study protocol. 

### 4.4. Sample Size 

Sample size calculation (Alpha = 0.05; Power = 95%) for two independent study groups and a continuous primary endpoint required 30 total participants for a split-mouth design. A total of 30 patients were visited before the beginning of the study, and all of them agreed to participate and complete the study. 

The following mathematical formula was used for sample size calculation:

$$\mathrm{Sample size}\;=\;\frac{Z_{\left(1-\frac{\alpha }{2}\right)}^{2}p\left(1-p\right)}{d^{2}}$$

where $z_{\left(1-\frac{\alpha }{2}\right)}$ is the standard normal variate corresponding to 1.96 at 5% type 1 error, *p* is the expected proportion in population expressed as decimal and based on previous studies, and finally, *d* is the confidence level decided by the researcher and expressed as a decimal.

Concerning the variable Probing Depth, an expected mean of 6.13 mm was hypothesized, with a standard deviation of 0.54. The expected difference between the means was supposed to be 0.5; therefore, 30 patients were required [46]. 

### 4.5. Randomization and Blinding

Using a block randomization table, the data analyst generated a randomization sequence, considering a permuted block of 30 participants. Based on previously prepared sequentially numbered, opaque, sealed envelopes (SNOSE), an operator executed the professional oral hygiene procedures and then assigned quadrants to the respective treatment. For the domiciliary oral hygiene procedures, the two gels were concealed. Neither the operator nor the patients were aware of the treatment administered considering that no differences occurred between the two solutions. Even the data analyst was blinded for the allocation. For the domiciliary protocol, according to the split-mouth design, the two syringes had different colors to facilitate the participants during the domiciliary procedure, and on the package, it was written where the gel had to be applied to avoid mistakes.

### 4.6. Statistical Methods

Data were analyzed with R Software (R version 3.1.3, R Development Core Team, R Foundation for Statistical Computing, Wien, Austria). Descriptive statistics (mean, standard deviation, minimum, median, and maximum) were calculated for each group and variable. Data normality of the distributions was calculated with the Kolmogorov–Smirnov test. Subsequently, the Friedman test for repeated measures was applied, followed by the post hoc Dunn test in case the results were significant. 

Significance was predetermined as *p* < 0.05 for all the tests performed. 

## 5. Conclusions

At least for the first six months of non-surgical periodontal therapy, the experimental gel tested (containing postbiotics, lactoferrin, and Aloe Barbadensis Leaf Juice Powder) showed no significant differences compared with a conventional chlorhexidine gel for the periodontal parameters assessed in the present report. Accordingly, postbiotics are supposed to be a natural alternative with respect to traditional chemical substances like chlorhexidine. However, in order to guarantee an external validity of the results here obtained, further studies, conducted on the specific compounds of the gels instead of the final commercialized products, are required.

## Figures and Tables

**Table 1:** Descriptive statistics of probing pocket depth measurements (PPD).

Group	Time	Mean (*)	St Dev	Min	Median	Max
Control (CHX)	T0	6.37 ^a^	1.53	4.00	6.00	10.00
	T1	5.17 ^a^	1.38	2.00	5.00	9.00
	T2	4.28 ^a,b,c^	1.31	2.00	4.00	9.00
	T3	3.87 ^c^	1.11	2.00	4.00	7.00
Test (BPI)	T0	6.40 ^a^	1.53	4.00	6.00	10.00
	T1	5.15 ^a,b^	1.44	3.00	5.00	9.00
	T2	4.24 ^b,c^	1.30	2.00	4.00	9.00
	T3	3.76 ^c^	1.21	2.00	4.00	7.00

* Means with the same letters are not significantly different (*p* > 0.05). a,b,c: Intergroup and intragroup comparisons.

**Table 2:** Descriptive statistics of recession (R).

Group	Time	Mean (*)	St Dev	Min	Median	Max
Control (CHX)	T0	1.78 ^a^	1.55	0.00	2.00	6.00
	T1	2.07 ^a,b^	1.76	0.00	2.00	6.00
	T2	2.18 ^a,b,c^	1.84	0.00	2.00	7.00
	T3	2.15 ^c,d^	1.85	0.00	2.00	7.00
Test (BPI)	T0	1.54 ^a^	1.38	0.00	1.00	5.00
	T1	1.74 ^a,b,c^	1.44	0.00	2.00	6.00
	T2	1.76 ^c,d^	1.49	0.00	2.00	6.00
	T3	1.72 ^d^	1.45	0.00	2.00	6.00

* Means with the same letters are not significantly different (*p* > 0.05). a,b,c,d: Intergroup and intragroup comparisons.

**Table 3:** Descriptive statistics of dental mobility.

Group	Time	Mean (*)	St Dev	Min	Median	Max
Control (CHX)	T0	0.50 ^a^	0.80	0.00	0.00	3.00
	T1	0.50 ^a^	0.80	0.00	0.00	3.00
	T2	0.38 ^a^	0.55	0.00	0.00	2.00
	T3	0.39 ^a^	0.56	0.00	0.00	2.00
Test (BPI)	T0	0.47 ^a^	0.76	0.00	0.00	2.00
	T1	0.41 ^a^	0.67	0.00	0.00	2.00
	T2	0.31 ^a^	0.47	0.00	0.00	1.00
	T3	0.28 ^a^	0.46	0.00	0.00	1.00

* Means with the same letters are not significantly different (*p* > 0.05). a: Intergroup and intragroup comparisons.

**Table 4:** Descriptive statistics of bleeding on probing (BoP%).

Group	Time	Mean (*)	St Dev	Min	Median	Max
Control (CHX)	T0	0.35 ^a^	0.25	0.08	0.28	1.00
	T1	0.20 ^a,b^	0.16	0.00	0.18	0.55
	T2	0.14 ^b,c^	0.12	0.01	0.09	0.40
	T3	0.12 ^c,d^	0.11	0.01	0.07	0.36
Test (BPI)	T0	0.36 ^a^	0.26	0.04	0.34	1.00
	T1	0.17 ^b,c^	0.13	0.00	0.14	0.48
	T2	0.11 ^c,d^	0.09	0.01	0.08	0.37
	T3	0.09 ^d^	0.08	0.00	0.05	0.29

* Means with the same letters are not significantly different (*p* > 0.05). a,b,c,d: Intergroup and intragroup comparisons.

**Table 5:** Descriptive statistics of the plaque control record (PCR%).

Group	Time	Mean (*)	St Dev	Min	Median	Max
Control (CHX)	T0	0.85 ^a^	0.19	0.36	0.95	1.00
	T1	0.52 ^a,b^	0.19	0.13	0.50	1.00
	T2	0.40 ^b,c^	0.18	0.05	0.40	0.88
	T3	0.30 ^d^	0.15	0.04	0.31	0.75
Test (BPI)	T0	0.85 ^a^	0.19	0.36	0.94	1.00
	T1	0.50 ^b,c^	0.17	0.10	0.50	0.88
	T2	0.37 ^c,d^	0.18	0.00	0.41	0.75
	T3	0.28 ^d^	0.16	0.06	0.27	0.70

* Means with the same letters are not significantly different (*p* > 0.05). a,b,c,d: Intergroup and intragroup comparisons.

**Table 6:** Inclusion and exclusion criteria.

Inclusion criteria	Age between 18 and 70 years Presence of periodontal disease according to the recent 2017 classification (2017 World Workshop on the Classification of Periodontal and Peri-Implant Diseases and Conditions) [42]: severity from grade I onwards (grade I: clinical attachment loss of 1–2 mm, bone loss in the cervical third greater than 15%, no elements loss for periodontitis); complexity from grade II onwards (grade II: maximum probing depth of 5 mm, bone loss mainly horizontally) Presence of bilateral periodontal probes, both to the right and to the left of the midline incisal line Electric toothbrush and a floss/brush according to the dimensions of the interdental spaces. Written informed consent to take part of the study
Exclusion criteria	Cardiac pacemaker Psychological, neurological or psychiatric disorders Pregnancy or breastfeeding within the last 12 months Low compliance or inconsistent motivation Use of drugs or alcohol Antibiotic therapy in the previous 6 months Presence of periodontal probes exclusively on the right or left side compared to the midline incisal line

**Table 7:** Products used in the study.

Gel	Manufacturer	Composition
Biorepair Parodontgel Intensive	Coswell S.p.A., Funo di Argelato, BO, Italy	Aqua, Propylene Glycol, Peg-40 Hydrogenated Castor Oil, Xylitol, Xanthan Gum, Silica, Zinc Hydroxyapatite, Zinc PCA, Aloe Barbadensis Leaf Juice Powder, *Lactobacillus Ferment*, Sodium Hyaluronate, Lactoferrin, Solidago Virgaurea Extract, Aroma, Sodium Benzoate, Phenylpropanol, Benzyl Alcohol, Hydroxyacetophenone, Sodium Saccharin, *O*-Cymen-5-ol, Mannitol, Decylene Glycol, Sodium Myristoyl Sarcosinate, Sodium Methyl Cocoyl Taurate, Citric Acid, Potassium Sorbate, Phenoxyethanol, Linalool, Benzyl Benzoate, Limonene.
Curasept Periodontal Gel (with 1% chlorhexidine)	Curasept S.p.A, Saronno, VA, Italy	Purified water, Propylene glycol, Hydroxy Ethyl Cellulose, PVP/VA copolymer, PEG-40 hydrogenated castor oil, Chlorhexidine digluconate, Sodium acetate, Aroma, Acetic acid, Sodium metabisulfite, Ascorbic acid

**Table 8:** Protocol adopted for the study.

Appointment	Procedures
	Signature to the informed consent for the study
	Assessment of periodontal clinical indices
	Professional supragingival and subgingival oral hygiene
Baseline (T0)	Supragingival and subgingival decontamination with glycine powders. Group 1: application in periodontal pockets of quadrants Q1 and Q3 of Biorepair Parodontgel Intensive; irrigation of periodontal pockets of quadrants Q2 and Q4 with chlorhexidine 1% gel. Group 2: irrigation of periodontal pockets of quadrants Q1 and Q3 with Curasept Periodontal Gel with 1% chlorhexidine; application in periodontal pockets of quadrants Q2 and Q4 of Biorepair Parodontgel Intensive. Domiciliary use of the two gels for the same quadrants until the 14 following days from the visit.
After 1 month (T1) After 3 months (T2) After 6 months (T3)	Assessment of periodontal clinical indices Professional supragingival and subgingival oral hygiene Group 1: application in periodontal pockets of quadrants Q1 and Q3 of Biorepair Parodontgel Intensive; irrigation of periodontal pockets of quadrants Q2 and Q4 with chlorhexidine 1% gel. Group 2: irrigation of periodontal pockets of quadrants Q1 and Q3 with Curasept Periodontal Gel with 1% chlorhexidine; application in periodontal pockets of quadrants Q2 and Q4 of Biorepair Parodontgel Intensive.
	Domiciliary use of the two gels for the same quadrants until the 14 following days from the visit.

## Data Availability

Data are available upon reasonable request from the corresponding author.

## References

[1] Baelum V., Lopez R. (2013). Periodontal disease epidemiology—Learned and unlearned?. Periodontol. 2000.

[2] Preshaw P.M., Alba A.L., Herrera D., Jepsen S., Konstantinidis A., Makrilakis K., Taylor R. (2012). Periodontitis and diabetes: A two-way relationship. Diabetologia.

[3] Kinane D.F., Chestnutt I.G. (2000). Smoking and periodontal disease. Crit. Rev. Oral Biol. Med..

[4] Wilton J.M., Griffiths G.S., Curtis M.A., Maiden M.F., Gillett I.R., Wilson D.T., Sterne J.A., Johnson N.W. (1988). Detection of high-risk groups and individuals for periodontal diseases. Systemic predisposition and markers of general health. J. Clin. Periodontol..

[5] Barr C., Lopez M.R., Rua-Dobles A. (1992). Periodontal changes by HIV serostatus in a cohort of homosexual and bisexual men. J. Clin. Periodontol..

[6] Shapira L., Wilensky A., Kinane D.F. (2005). Effect of genetic variability on the inflammatory response to periodontal infection. J. Clin. Periodontol..

[7] Marsh P.D. (2005). Dental plaque: Biological significance of a biofilm and community life-style. J. Clin. Periodontol..

[8] Berezow A.B., Darveau R.P. (2011). Microbial shift and periodontitis. Periodontol. 2000.

[9] Mombelli A. (2018). Microbial colonization of the periodontal pocket and its significance for periodontal therapy. Periodontol. 2000.

[10] Feres M. (2008). Antibiotics in the treatment of periodontal diseases: Microbiological basis and clinical applications. Ann. R Australas. Coll. Dent. Surg..

[11] Meimandi M., Talebi Ardakani M.R., Esmaeil Nejad A., Yousefnejad P., Saebi K., Tayeed M.H. (2017). The Effect of Photodynamic Therapy in the Treatment of Chronic Periodontitis: A Review of Literature. J. Lasers Med. Sci..

[12] Butera A., Gallo S., Pascadopoli M., Luraghi G., Scribante A. (2021). Ozonized Water Administration in Peri-Implant Mucositis Sites: A Randomized Clinical Trial. Appl. Sci..

[13] Invernici M.M., Salvador S.L., Silva P., Soares M., Casarin R., Palioto D.B., Souza S., Taba M., Novaes A.B., Furlaneto F. (2018). Effects of Bifidobacterium probiotic on the treatment of chronic periodontitis: A randomized clinical trial. J. Clin. Periodontol..

[14] Butera A., Pascadopoli M., Gallo S., Lelli M., Tarterini F., Giglia F., Scribante A. (2021). SEM/EDS Evaluation of the Mineral Deposition on a Polymeric Composite Resin of a Toothpaste Containing Biomimetic Zn-Carbonate Hydroxyapatite (microRepair^®^) in Oral Environment: A Randomized Clinical Trial. Polymers.

[15] Francino M. (2016). Antibiotics and the human gut microbiome: Dysbioses and accumulation of resistances. Front. Microbiol..

[16] Joint Food and Agriculture Organization (FAO)/World Health Organization (WHO) Working Group (2002). Working Group Report on Drafting Guidelines for the Evaluation of Probiotics in Food.

[17] Ince G., Gürsoy H., Ipçi S.D., Cakar G., Emekli-Alturfan E., Yılmaz S. (2015). Clinical and Biochemical Evaluation of Lozenges Containing Lactobacillus reuteri as an Adjunct to Non-Surgical Periodontal Therapy in Chronic Periodontitis. J. Periodontol..

[18] Żółkiewicz J., Marzec A., Ruszczyński M., Feleszko W. (2020). Postbiotics-A Step Beyond Pre- and Probiotics. Nutrients.

[19] Piepho H.P. (2004). An Algorithm for a Letter-Based Representation of All-Pairwise Comparisons. J. Comput. Graph. Stat..

[20] Scribante A., Poggio C., Gallo S., Riva P., Cuocci A., Carbone M., Arciola C.R., Colombo M. (2020). In Vitro Re-Hardening of Bleached Enamel Using Mineralizing Pastes: Toward Preventing Bacterial Colonization. Materials.

[21] Belibasakis G.N., Mylonakis E. (2015). Oral infections: Clinical and biological perspectives. Virulence.

[22] Lutovac M., Popova O.V., Macanovic G., Kristina R., Lutovac B., Ketin S., Biocanin R. (2017). Testing the Effect of Aggressive Beverage on the Damage of Enamel Structure. Open Access Maced. J. Med. Sci..

[23] Colombo M., Poggio C., Lasagna A., Chiesa M., Scribante A. (2019). Vickers Micro-Hardness of New Restorative CAD/CAM Dental Materials: Evaluation and Comparison after Exposure to Acidic Drink. Materials.

[24] Deng Z.L., Szafrański S.P., Jarek M., Bhuju S., Wagner-Döbler I. (2017). Dysbiosis in chronic periodontitis: Key microbial players and interactions with the human host. Sci. Rep..

[25] Ikram S., Hassan N., Raffat M.A., Mirza S., Akram Z. (2018). Systematic review and meta- analysis of double- blind, placebo- controlled, randomized clinical trials using probiotics in chronic periodontitis. J. Investig. Clin. Dent..

[26] Vivekananda M.R., Vandana K.L., Bhat K.G. (2010). Effect of the probiotic *Lactobacilli reuteri* (Prodentis) in the management of periodontal disease: A preliminary randomized clinical trial. J. Oral Microbiol..

[27] Teughels W., Durukan A., Ozcelik O., Pauwels M., Quirynen M., Haytac M.C. (2013). Clinical and microbiological effects of *Lactobacillus reuteri* probiotics in the treatment of chronic periodontitis: A randomized placebo- controlled study. J. Clin. Periodontol..

[28] Szkaradkiewicz A.K., Stopa J., Karpiński T.M. (2014). Effect of oral administration involving a probiotic strain of *Lactobacillus reuteri* on pro- inflammatory cytokine response in patients with chronic periodontitis. Arch. Immunol. Ther. Exp..

[29] Varoni E., Tarce M., Lodi G., Carrassi A. (2012). Chlorhexidine (CHX) in dentistry: State of the art. Minerva Stomatol..

[30] Ishikawa K.H., Bueno M.R., Kawamoto D., Simionato M.R.L., Mayer M.P.A. (2021). Lactobacilli postbiotics reduce biofilm formation and alter transcription of virulence genes of *Aggregatibacter actinomycetemcomitans*. Mol. Oral Microbiol..

[31] Park E., Ha J., Lim S., Kim G., Yoon Y. (2021). Development of postbiotics by whey bioconversion with *Enterococcus faecalis* M157 KACC81148BP and *Lactococcus lactis* CAU2013 KACC81152BP for treating periodontal disease and improving gut health. J. Dairy Sci..

[32] Izuddin W.I., Humam A.M., Loh T.C., Foo H.L., Samsudin A.A. (2020). Dietary Postbiotic Lactobacillus plantarum Improves Serum and Ruminal Antioxidant Activity and Upregulates Hepatic Antioxidant Enzymes and Ruminal Barrier Function in Post-Weaning Lambs. Antioxidants.

[33] Osman A., El-Gazzar N., Almanaa T.N., El-Hadary A., Sitohy M. (2021). Lipolytic Postbiotic from *Lactobacillus paracasei* Manages Metabolic Syndrome in Albino Wistar Rats. Molecules.

[34] Butera A., Gallo S., Maiorani C., Preda C., Chiesa A., Esposito F., Pascadopoli M., Scribante A. (2021). Management of Gingival Bleeding in Periodontal Patients with Domiciliary Use of Toothpastes Containing Hyaluronic Acid, Lactoferrin, or Paraprobiotics: A Randomized Controlled Clinical Trial. Appl. Sci..

[35] Yadav N., Lamba A.K., Thakur A., Faraz F., Tandon S., Pahwa P. (2014). Effect of periodontal therapy on lactoferrin levels in gingival crevicular fluid. Aust. Dent. J..

[36] Berlutti F., Pilloni A., Pietropaoli M., Polimeni A., Valenti P. (2011). Lactoferrin and oral diseases: Current status and perspective in periodontitis. Ann. Stomatol..

[37] Yeturu S.K., Acharya S., Urala A.S., Pentapati K.C. (2016). Effect of *Aloe vera*, chlorine dioxide, and chlorhexidine mouth rinses on plaque and gingivitis: A randomized controlled trial. J. Oral Biol. Craniofac. Res..

[38] Moghaddam A.A., Radafshar G., Jahandideh Y., Kakaei N. (2017). Clinical evaluation of effects of local application of *Aloe vera* gel as an adjunct to scaling and root planning in patients with chronic periodontitis. J. Dent..

[39] Ipshita S., Kurian I.G., Dileep P., Kumar S., Singh P., Pradeep A.R. (2018). One percent alendronate and *Aloe vera* gel local host modulating agents in chronic periodontitis patients with class II furcation defects: A randomized, controlled clinical trial. J. Investig. Clin. Dent..

[40] Kurian I.G., Dileep P., Ipshita S., Pradeep A.R. (2018). Comparative evaluation of subgingivally-delivered 1% metformin and *Aloe vera* gel in the treatment of intrabony defects in chronic periodontitis patients: A randomized, controlled clinical trial. J. Investig. Clin. Dent..

[41] Sánchez M., González-Burgos E., Iglesias I., Gómez-Serranillos M.P. (2020). Pharmacological Update Properties of *Aloe vera* and its Major Active Constituents. Molecules.

[42] Berglundh T., Armitage G., Araujo M.G., Avila-Ortiz G., Blanco J., Camargo P.M., Chen S., Cochran D., Derks J., Figuero E. (2018). Peri-implant diseases and conditions: Consensus report of workgroup 4 of the 2017 World Workshop on the Classification of Periodontal and Peri-Implant Diseases and Conditions. J. Clin. Periodontol..

[43] Lang N.P., Joss A., Orsanic T., Gusberti F.A., Siegrist B.E. (1986). Bleeding on probing. A predictor for the progression of periodontal disease?. J. Clin. Periodontol..

[44] O’Leary T.J., Drake R.B., Naylor J.E. (1972). The Plaque Control Record. J. Periodontol..

[45] Purkait S., Bandyopadhyaya P., Mallick B., Das I. (2016). Classification of tooth mobility—Concept Revisited. Int. J. Rec. Adv. Multidiscip. Res..

[46] Sreedhar A., Sarkar I., Rajan P., Pai J., Malagi S., Kamath V., Barmappa R. (2015). Comparative evaluation of the efficacy of curcumin gel with and without photo activation as an adjunct to scaling and root planing in the treatment of chronic periodontitis: A split mouth clinical and microbiological study. J. Nat. Sci. Biol. Med..

